# Stepwise Fabrication of Co-Embedded Porous Multichannel Carbon Nanofibers for High-Efficiency Oxygen Reduction

**DOI:** 10.1007/s40820-019-0264-2

**Published:** 2019-04-06

**Authors:** Zeming Tang, Yingxuan Zhao, Qingxue Lai, Jia Zhong, Yanyu Liang

**Affiliations:** 1 Jiangsu Key Laboratory of Materials and Technology for Energy Conversion, College of Materials Science and Technology, Nanjing University of Aeronautics and Astronautics, Nanjing, 210016 People’s Republic of China; 2 Jiangsu Collaborative Innovation Center for Advanced Inorganic Function Composites, Nanjing, 211816 People’s Republic of China

**Keywords:** Nonprecious metal material, Multichannel carbon nanofiber, Oxygen reduction reaction, Core–shell nanoparticle, Synergistic effect

## Abstract

A novel nonprecious metal material consisting of Co-embedded porous interconnected multichannel carbon nanofibers (Co/IMCCNFs) was rationally designed for oxygen reduction reaction (ORR) electrocatalysis. In the synthesis, ZnCo_2_O_4_ was employed to form interconnected mesoporous channels and provide highly active Co_3_O_4_/Co core–shell nanoparticle-based sites for the ORR. The IMC structure with a large synergistic effect of the N and Co active sites provided fast ORR electrocatalysis kinetics. The Co/IMCCNFs exhibited a high half-wave potential of 0.82 V (vs. reversible hydrogen electrode) and excellent stability with a current retention up to 88% after 12,000 cycles in a current–time test, which is only 55% for 30 wt% Pt/C.

**Electronic supplementary material:**

The online version of this article (10.1007/s40820-019-0264-2) contains supplementary material, which is available to authorized users.

## Highlights

- An interconnected structure is developed by evaporation of zinc species using a ZnCo_2_O_4_ precursor as the cobalt resource, enabling communications between channels as well as homogeneous loading of active sites.
- A shell structure of Co_3_O_4_ is formed on the surface of a zero-valent Co^0^ core during a stepwise carbothermic reduction of ZnCo_2_O_4_.
- The Co-embedded multichannel carbon nanofibers exhibit not only a superior half-wave potential, but also an excellent durability compared to those of the commercial 30% Pt/C.

**Electronic supplementary material:**

The online version of this article (10.1007/s40820-019-0264-2) contains supplementary material, which is available to authorized users.

## Graphical abstract

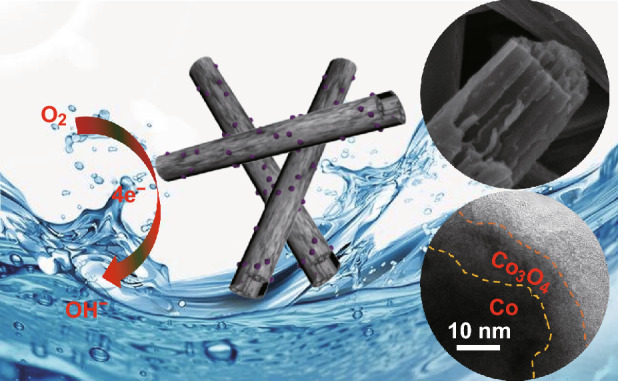

## Introduction

Fuel cells are considered as ideal alternatives to fossil fuels owing to their high energy conversion efficiencies and environmental friendliness. An H_2_–O_2_ fuel cell exhibits a thermodynamic efficiency of approximately 80% at 25 °C, significantly higher than that of an internal combustion engine (10–20%) [1–3]. Nevertheless, the sluggish kinetics of the oxygen reduction reaction (ORR) on the cathode hinder the large-scale industrial application of the fuel cells [4, 5]. Currently, Pt and Pt-based alloys exhibit superior electrocatalytic properties in acid and alkaline media. However, owing to the high costs and unsatisfactory stabilities of Pt and Pt-based alloys, it is required to develop cheap efficient ORR catalysts [6–9].

Transition metal-based materials, such as transition metal and metal oxides/carbides/nitrides, are ideal ORR alternatives to Pt-based precious metal materials [10–13]. The transition metal-based materials exhibit very high catalytic activities, long-term durabilities, and high tolerances to the crossover effect by methanol for the ORR owing to their low activation energies for the absorption and dissociation of O_2_ [14, 15]. Although these candidates exhibit reasonable ORR activities, the low conductivities and unsatisfactory agglomeration hinder their application as ORR catalysts. Extensive studies have been carried out to combine transition metals with carbon materials to enhance the electric conductivity as well as the dispersion of metal-based active sites [16, 17]. Moreover, theoretical calculations suggested that dopants of heteroatoms in the *sp*^2^ lattice of graphitic carbon can turn the oxygen adsorption mode into diatomic adsorption, which changes the electron cloud of the graphite carbon and significantly enhances the kinetics of the ORR [18–20].

Carbon nanotubes (CNTs), one-dimensional graphitized carbon structures with a high suppleness, satisfactory specific surface area, and high electrical conductivity, have been widely employed in gas adsorption, electrocatalysis, energy storage, and conversion applications [21]. Unlike CNTs, which are regularly composed of graphene layers rolled up into circular tubes with flats parallel to the vertical axis, carbon nanofibers (CNFs) can be engineered into various morphologies such as dendritic, core–shell, and hollow structures, particularly by electrospinning [22–26]. In addition, CNFs with parallel channels are promising for electrocatalysis applications owing to the high exposure as well as utilization of active sites in the fibrous carbon skeleton. Recently, Kim et al. [27] have employed the electrospinning strategy to fabricate multichannel CNFs (MCCNFs) using the thermal stability difference between polyacrylonitrile (PAN) and poly(methyl methacrylate) (PMMA). Similarly, David et al. [28] fabricated parallel channels in CNFs by a controllable decomposition of a dispersion phase of polystyrene (PS) in PAN for Li–S battery applications. The generation of parallel channels within the skeleton of nanofibers (NFs) significantly increases the specific surface area. However, the disconnection among the channels limits the transmission of electrons and mass during the ORR electrocatalysis, which is addressed by developing interconnected multichannel (IMC) structures for CNF-based ORR electrocatalysts.

In this study, Co-embedded interconnected porous multichannel carbon nanofibers (Co/IMCCNFs) were fabricated through the electrospinning strategy. During the synthesis process, zinc salt was added into polymeric precursors of PS and PAN, yielding interconnected porous MCCNFs after pyrolysis at 950 °C. To further improve the ORR catalytic activity of the MCCNFs, the electrochemically active cobalt was efficiently incorporated into the MCCNFs using ZnCo_2_O_4_ as the intermediate. Owing to the interconnected porous frameworks as well as the improved electron and mass transmission pathways, the Co/IMCCNFs exhibited a satisfactory half-wave potential and excellent electrochemical durability for the ORR.

## Experimental Methods

### Chemicals

PAN (molecular weight (MW) = 210,000) was purchased from Goodfellow Cambridge Limited. PS (MW = 28,000) particles were obtained from Aladdin Ltd. Zinc (II) acetylacetonate (Zn(acac)_2_) and cobalt (II) acetylacetonate (Co(acac)_2_) were purchased from Alfa Aesar Ltd. Ammonium hydroxide (NH_3_·H_2_O), N, N’-dimethylformamide (DMF), and ethanol absolute (EtOH) were purchased from Sinopharm Chemical Reagent Co. All reagents were used without further purification.

### Preparation of Co/CNFs

For the synthesis of Co/CNFs, 500 mg of PS and 400 mg of Zn(acac)_2_ were blended into 10 mL of DMF to obtain a homogeneous solution under stirring for 12 h at room temperature. The PS/Zn(acac)_2_ solution was then electrospun into NFs at a high voltage of 10 kV and appropriate collection distance of 15 cm. The collected fibers were then transferred into a vacuum drying oven at 60 °C for 24 h. In the second step, spinel ZnCo_2_O_4_ was employed as the intermediate to incorporate cobalt. Typically, 0.319 g of Co(acac)_2_ and 0.163 g of Zn(acac)_2_ were dissolved in 96 mL of ethanol and 4 mL of distilled water. 0.494 g of fibers preoxidized at 250 °C in air were added to the above solution, followed by the addition of 1 mL of NH_3_·H_2_O. The reaction was carried out at 60 °C under stirring for 2 h. Subsequently, the reaction mixture was transferred to a 100-mL autoclave for a solvothermal reaction at 150 °C for 3 h. The resulting product was collected by filtration, washed several times with ethanol and water, and then dried in vacuum at 60 °C. Finally, the obtained product was heated in Ar at 950 °C for 4 h; the heating rate was 5 °C min^−1^.

### Preparation of Co/IMCCNFs

The Co/IMCCNFs were prepared by the same method as that for the Co/CNFs except the addition of 500 mg of PAN in the PS/Zn(acac)_2_ solution.

### Preparation of Co/MCCNFs-D

PS (500 mg), PAN (500 mg), and Co(acac)_2_ (400 mg) were blended into 10 mL of DMF to obtain a homogeneous solution. The PS/PAN/Co(acac)_2_ solution was then electrospun into NFs under the same conditions. The collected fibers were dried in the vacuum oven at 60 °C for 24 h. In the second step, the fibers were preoxidized at 250 °C in air and then pyrolyzed at 950 °C for 4 h in Ar (the heating rate was 5 °C min^−1^).

### Characterization

The physical properties of the prepared samples were analyzed by X-ray diffraction (XRD, SIEMENS Diffractometer D5000 with a Cu K_α_ radiation source), X-ray photoelectron spectroscopy (XPS, ESCALab 220i-XL electron spectrometer), field emission scanning electron microscopy (FE-SEM, ULTRA-55), transmission electron microscopy (TEM, JSM-2100), and Brunauer–Emmett–Teller (BET) measurement (3H-2000PS1/2 static volume method, China).

### Electrochemical Measurements

The electrochemical characteristics were measured in a three-electrode system with a glass carbon loading with the electrocatalyst (0.25 mg cm^−2^) as the working electrode, platinum sheet as the counter electrode, and saturated calomel electrode (SCE) as the reference electrode. Cyclic voltammetry (CV) and linear sweep voltammetry tests of a rotating disk electrode (RDE) and rotating ring–disk electrode (RRDE) were carried out in an O_2_-saturated 0.1 M KOH electrolyte at room temperature using a CHI760D electrochemical workstation equipped with a modulated speed electrode rotator (Pine Research Instrumentation). The tested electrode potential (*E* (SCE)) was calibrated to the reversible hydrogen electrode potential (*E* (RHE)) according to the equation: *E* (RHE) = *E* (SCE) + 0.059 × pH + 0.241 [29, 30].

For the RRDE measurement, the ring potential was set at 1.4 V (vs. RHE). %HO_2_^−^ and electron transfer number (*n*) were evaluated by Eqs. (1) and (2):

1 $$\% {\text{HO}}_{2}^{ - } = 200 \times \frac{{I_{\text{r}} /N}}{{I_{\text{d }} + I_{\text{r}} /N}}$$

2 $$n = 4 \times \frac{{I_{\text{d}} }}{{I_{\text{d }} + I_{\text{r}} /N}}$$

where *I*_d_ is the disk current, *I*_r_ is the ring current, and *N* (= 0.37) is the current collection efficiency of the Pt ring.

## Results and Discussion

**Fig. 1:**
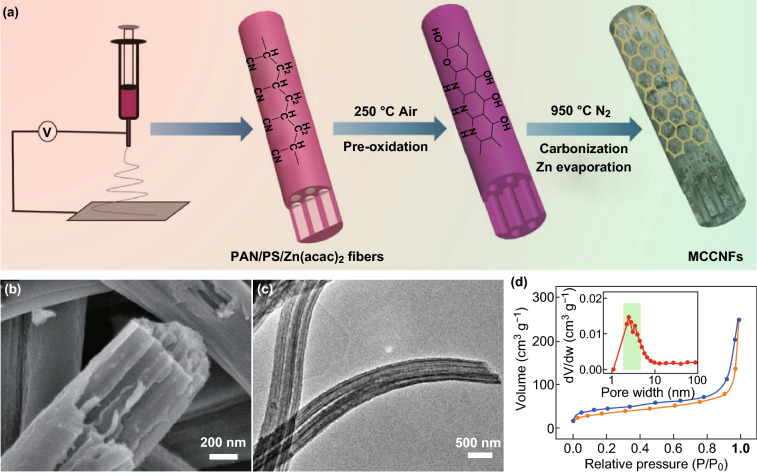
**a** Schematic of the formation of interconnected channels in the MCCNFs. **b, c** SEM and TEM images of the MCCNFs. **d** N_2_ adsorption and desorption isotherms of the porous MCCNFs (inset: pore size distribution of the porous MCCNFs)

The MCCNFs with an interconnected structure were fabricated by electrospinning using the polymer mixture of PS and PAN. The differential scanning calorimetry and thermogravimetric curves in Fig. S1 confirm that PS begins to decompose at 289.1 °C and completely decomposes at 423.1 °C, achieving MC structures in the carbon skeleton. Furthermore, in order to improve the connection properties between the parallel channels for enhanced oxygen reduction kinetics, extra zinc salt was incorporated into the polymer mixture. After preoxidation at 250 °C followed by a high-temperature carbonization at 950 °C, the metallic zinc formed by the carbothermic reduction was completely evaporated, leading to IMCs in the skeleton of the CNFs, as shown in Fig. 1a.

SEM and TEM analyses were performed to investigate the structures of the porous MCCNFs. The parallel channels with diameters of approximately 60 nm were successfully fabricated; no residual zinc-containing particles were observed on the surfaces of the NFs (Fig. 1b, c). A BET measurement confirmed that abundant mesoporous structures (with sizes of approximately 3.5 nm) were successfully generated in the porous MCCNFs owing to the reduction and evaporation of zinc species (Fig. 1d). Such unique IMC structures with enhanced mass transfer kinetics and exposed surfaces can be not only a metal-free ORR electrocatalyst but also an ideal support to construct transition metal-loaded nonprecious metal ORR electrocatalysts.

**Fig. 2:**
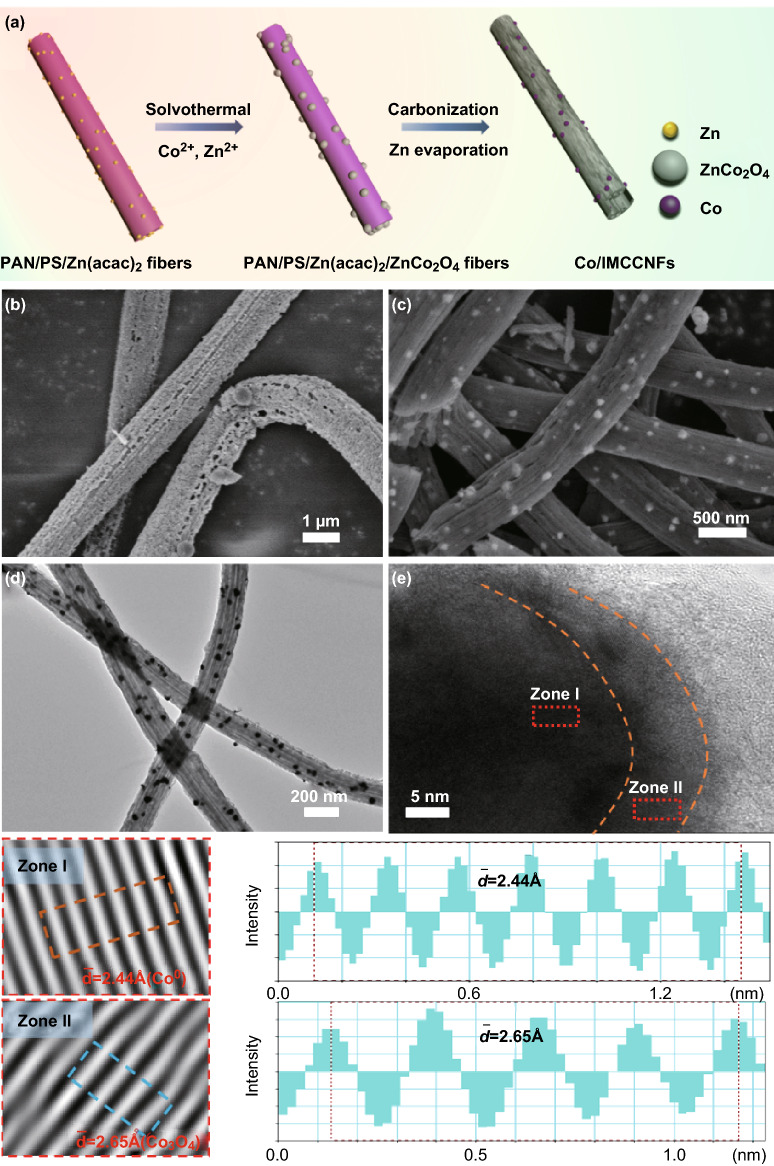
**a** Schematic of the synthesis of the Co/IMCCNFs. **b, c** SEM images of the ZnCo_2_O_4_ NFs and Co/IMCCNFs. **d, e** TEM and HRTEM images of the Co/IMCCNFs (the bottom images show the lattice fringes and lattice spacing of the Co/IMCCNFs)

Highly active Co nanoparticles were then incorporated into the porous MCCNFs using ZnCo_2_O_4_ as the intermediate. Figure 2 shows a schematic of the synthesis and SEM and TEM images of the prepared Co/IMCCNFs. Precursor polymer NFs with a homogeneously dispersed zinc salt were prepared by electrospinning. After a hydrothermal reaction, a well-defined spinel ZnCo_2_O_4_ intermediate was grown on the surface (Fig. 2b). As shown in Fig. 2c, d, cobalt nanoparticles with diameters of approximately 50 nm were uniformly anchored on the porous MCCNFs. Compared to the MCCNFs directly prepared in one step without ZnCo_2_O_4_ as the intermediate (Co/MCCNFs-D), the Co/IMCCNFs exhibited a higher surface roughness (Fig. S2), which could be attributed to the reduction and evaporation of zinc species from the ZnCo_2_O_4_ intermediate. Furthermore, a core–shell structure can be observed in the high-resolution TEM (HRTEM) image in Fig. 2e, which confirms the formation of the shell structure of Co_3_O_4_ on the surface of the metallic Co^0^ core during the stepwise carbothermic reduction of ZnCo_2_O_4_. In addition, some graphitic carbon shells with a lattice spacing of 3.5 Å were generated on the cobalt-containing nanoparticles (Fig. S3), leading to an increased graphitization compared to that of the MCCNFs [31–34]. Such a stable nanostructure with double metal oxide and graphitic carbon shells is expected to provide excellent ORR electrocatalysis properties with high catalytic activity and stability [35, 36].

**Fig. 3:**
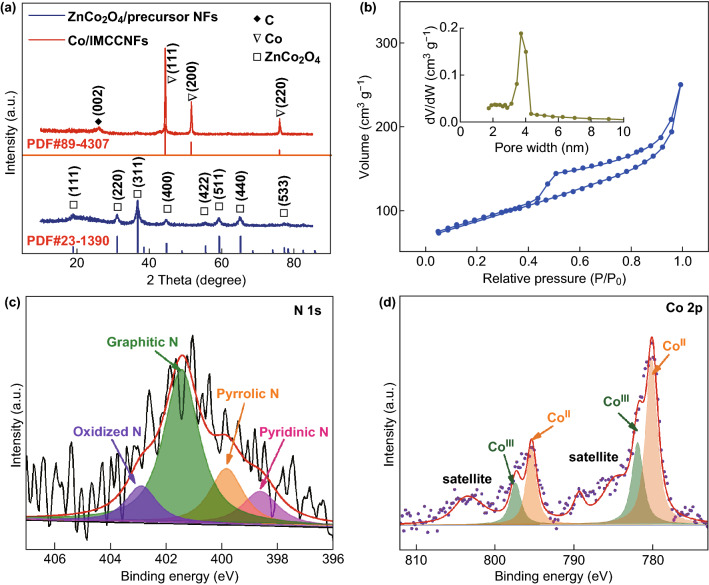
**a** Powder XRD pattern of the ZnCo_2_O_4_/precursor NFs and Co/IMCCNFs. **b** N_2_ adsorption and desorption isotherms of the Co/IMCCNFs (inset: pore size distribution of the Co/IMCCNFs). **c** N 1 *s* and **d** Co 2*p* XP spectra of the Co/IMCCNFs

The structures of the prepared ZnCo_2_O_4_/precursor NFs and Co/IMCCNFs were further analyzed by XRD measurements. Figure 3a shows a typical XRD pattern of the face-centered cubic ZnCo_2_O_4_ (PDF#23-1390). After the carbonization at 950 °C, high diffraction peaks are observed at 44°, 51°, and 75°, corresponding to the (1 1 1), (2 0 0), and (2 2 0) crystal planes of zero-valent cobalt (PDF #89-4307), respectively. Simultaneously, the diffraction peak of carbon is observed at 26°, while no extra peaks corresponding to zinc species are observed, suggesting the complete removal of zinc species by the carbothermic reduction and evaporation.

Further, nitrogen adsorption/desorption isotherms are shown in Fig. 3b. The prepared Co/IMCCNFs exhibited a large BET specific surface area of 298.5 m^2^ g^−1^. Moreover, they exhibited a typical mesoporous structure with pore sizes of approximately 3.5 nm. The mesoporous structure facilitates the channel interconnection and promotes the mass transfer toward the ORR [37, 38].

An XPS analysis was carried out to observe the surface electronic states and compositions of the products. In the high-resolution N 1 *s* XP spectrum in Fig. 3c, four peaks at 398.59, 399.79, 401.40, and 402.87 eV can be deconvoluted corresponding to pyridinic N, pyrrolic N, graphitic N, and oxidized N, respectively [39, 40]. Among the different types of N, the graphitic N is dominant, suggesting a stable C–N hexatomic ring originated from the cyanide moieties in the PAN skeleton during the preoxidation, which exists after the high-temperature pyrolysis. The high-resolution Co 2*p* XP spectrum in Fig. 3d shows two peaks at 780.56 and 795.43 eV, corresponding to the Co 2*p*_3/2_ and Co 2*p*_1/2_ states, respectively. Furthermore, the Co 2*p*_3/2_ signal can be deconvoluted to two different peaks centered at 780.15 and 781.92 eV, corresponding to Co (II) and Co (III), respectively [41–44]. These results further verify the existence of cobalt oxides on the surfaces of the Co/IMCCNFs, which is consistent with the HRTEM images.

The RDE voltammogram shows that the optimized interconnected channel structure can enhance the ORR electrocatalytic activity (half-wave potential or limiting current). However, it is still far from that of the commercial Pt/C catalyst (Fig. S4). Cobalt, regarded as a highly catalytic metal toward the ORR, was successfully incorporated into the IMC structure by a stepwise solvothermal growth and carbothermic reduction.

**Fig. 4:**
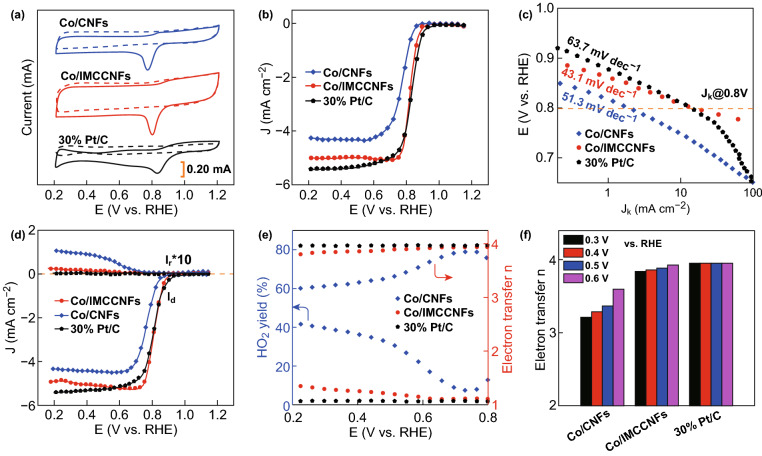
**a** CV curves of 30% Pt/C, Co/IMCCNFs, and Co/CNFs on glassy carbon electrodes in O_2_-saturated (solid curve) and N_2_-saturated (dashed curve) 0.1 M KOH electrolytes. **b, c** RDE voltammograms and corresponding Tafel plots of 30% Pt/C, Co/IMCCNFs, and Co/CNFs. **d** RRDE voltammograms recorded with Co/IMCCNFs and 30% Pt/C in the O_2_-saturated 0.1 M KOH at 1600 rpm. **e** Percentages of peroxide (lower line) and electron transfer numbers (*n*) (upper line) of 30% Pt/C, Co/IMCCNFs, and Co/CNFs calculated using the RRDE data in (**d**). **f** Electron transfer numbers of 30% Pt/C, Co/IMCCNFs, and Co/CNFs at potentials of 0.3, 0.4, 0.5, and 0.6 V (vs. RHE)

To assess the ORR catalytic activities, CV analyses were performed in O_2_- and N_2_-saturated 0.1 M KOH electrolytes. Remarkably, the Co/IMCCNFs exhibited a significantly larger positive ORR onset potential of 0.896 V (vs. RHE) and higher cathodic current, suggesting an enhanced ORR electrocatalysis through anchoring of the highly active cobalt sites on the unique MC structure. As shown in Fig. 4a, the Co/IMCCNFs exhibit a larger peak potential than that of the Co/CNFs, suggesting a lower overpotential for the ORR of the former structure. The RDE voltammograms show that the Co/IMCCNFs have a larger ORR onset potential and higher limiting current density (Fig. 4b) than those of the Co/CNFs. Moreover, the Co/IMCCNFs exhibit a high half-wave potential of 0.82 V, equal to that of the commercial 30% Pt/C (0.82 V) and larger than that of the Co/CNFs. These excellent electrocatalysis properties could be attributed to the highly active cobalt sites efficiently anchored on the MC structures, which not only ensure a fast mass transfer, but also significantly enhance the exposure and effective utilization of the Co active sites [45]. Moreover, the ORR performances of Co/IMCCNFs pyrolyzed at different temperatures (850, 950, and 1050 °C) during the synthesis were also evaluated. As shown in Fig. S6, the Co/IMCCNFs pyrolyzed at 950 °C exhibited the largest half-wave potential and limiting current among those of the three samples. When the sample was pyrolyzed at 850 °C, the connection between channels was reduced owing to the incomplete evaporation of zinc. On the other hand, the decrease in nitrogen content led to a decrease in the catalytic performance of the catalyst for the ORR with the increase in the pyrolysis temperature to 1050 °C.

The excellent ORR activity can also be verified through a Tafel diagram (Fig. 4c). The Co/CNFs exhibit a satisfactory Tafel slope of 51.3 mV dec^−1^, significantly lower than that of 30% Pt/C (63.7 mV dec^−1^) at the half-wave potential. Furthermore, the Co/IMCCNFs exhibit a superior Tafel slope of 43.1 mV dec^−1^, indicating the most favorable kinetic for the ORR among those of the considered samples [46–48]. As shown in Figs. 4d–f and S5, RRDE measurements were carried out to monitor the formation of peroxide species (HO_2_^−^) during the ORR [49–51]. The measured HO_2_^−^ yield for the Co/IMCCNFs in the potential range of 0.2–0.8 V (vs. RHE) is below ~ 10%, corresponding to a large electron transfer number of ~ 3.90, similar to that of 30% Pt/C (~ 3.96) but significantly larger than those of the Co/CNFs (~ 3.65), porous MCCNFs (~ 3.72), and Co/MCCNFs-D (~ 3.73). This indicates that the introduction of Co nanoparticles and MC structures significantly enhanced the four-electron process toward the ORR.

**Fig. 5:**
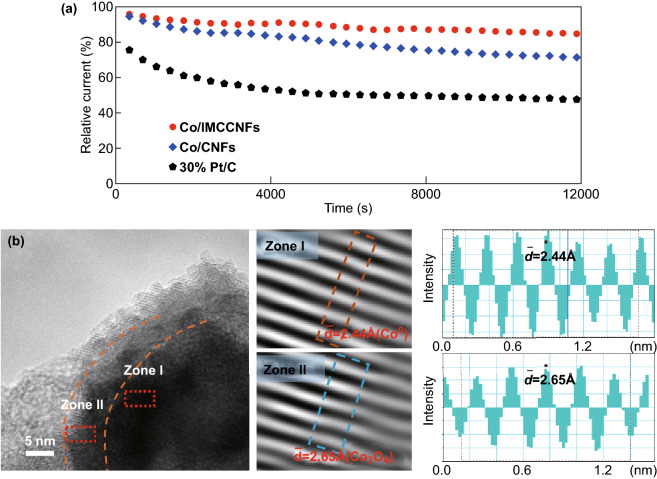
**a** *I*–*t* curves of 30% Pt/C, Co/IMCCNFs, and Co/CNFs. **b** High-resolution TEM images of the Co/IMCCNFs after the *I*–*t* test (the middle images show the lattice fringes, while the right patterns reveal the lattice spacing)

*I*–*t* tests were then carried out to evaluate the stabilities of the catalysts, as shown in Fig. 5a. The prepared Co/IMCCNF catalyst maintained up to 88% of the initial current after 12,000 cycles, which is a larger retention than that of 30% Pt/C (55%) [40]. To reveal the origin of the high stability of the Co/IMCCNFs, a high-resolution TEM analysis of the Co/IMCCNFs was performed after the *I*–*t* test. As shown in Fig. 5b, the integrated Co core–Co_3_O_4_ shell structure is well maintained after the *I*–*t* test. This can be attributed to the unique structure with the metal oxide shell, which is inactive in the alkaline solution and protects the Co core from corrosion, and graphitic degree-enhanced CNFs, which well maintained the excellent dispersion of Co active sites.

## Conclusion

We developed a stepwise strategy to synthesize novel CNFs with IMC structures anchored with Co_3_O_4_/Co core–shell nanoparticles as efficient catalysts for the ORR. Moreover, the incorporation of cobalt using the ZnCo_2_O_4_ intermediate further increased the mass loading of zinc, which promoted the connectivity of the MCCNFs in the subsequent pyrolysis process, leading to the larger specific surface area and faster mass transfer of the Co/IMCCNFs than those of the porous MCCNFs. Consequently, the Co/IMCCNFs exhibited a superior half-wave potential (~ 0.82 V), limiting current of ~ 5.08 mA cm^−2^, low HO_2_^−^ yield (below ~ 10%), large electron transfer number (~ 3.90), and satisfactory long-term durability. The proposed stepwise strategy paves the way for novel carbon fiber modification techniques for carbon-based nanomaterials for energy storage and conversion applications.

## Electronic supplementary material

Below is the link to the electronic supplementary material.

Supplementary material 1 (PDF 614 kb)
