# Supplementary material for: Stepwise Fabrication of Co-Embedded Porous Multichannel Carbon Nanofibers for High-Efficiency Oxygen Reduction
Source: Nanomicro Lett. 2019 Apr 6;11:33. doi: 10.1007/s40820-019-0264-2 (PMC7770944; doi:10.1007/s40820-019-0264-2)
Supplement: Supplementary file 1 — Supplementary material 1 (PDF 614 kb) [file 40820_2019_264_MOESM1_ESM.pdf]

Supporting Information for

Stepwise Fabrication of Co-Embedded Porous Multi-Channels Carbon Nanofibers for High-Efficiency Oxygen Reduction

Zeming Tang[1], Yingxuan Zhao[1], Qingxue Lai[1, *], Jia Zhong[1], Yanyu Liang[1, 2, *]

[1]Jiangsu Key Laboratory of Materials and Technology for Energy Conversion, College of Materials Science and Technology, Nanjing University of Aeronautics and Astronautics, Nanjing 210016, People's Republic of China

[2]Jiangsu Collaborative Innovation Center for Advanced Inorganic Function Composites, Nanjing 211816, People's Republic of China

*Corresponding authors. E-mail: laiqingxue@126.com (Qingxue Lai); liangyy403@126.com (Yanyu Liang)

Supplementary Figures

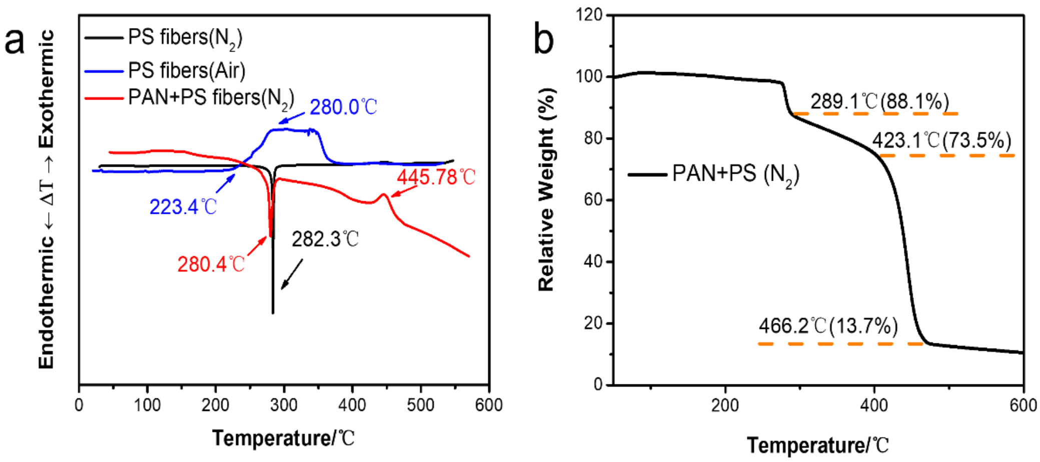

Fig. S1 a DSC curves of PS fibers (N_2), PS fibers (Air) and PAN+PS fibers (N_2) with the temperature range of 50-600 °C. **b** TGA curve of PAN+PS fibers (N_2) with the temperature range of 50-600 °C

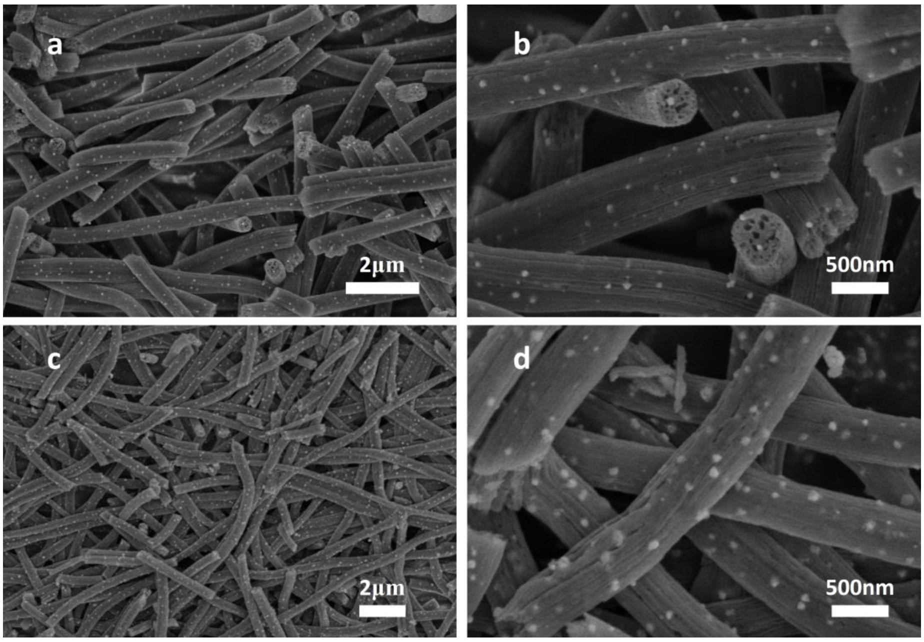

Fig. S2 SEM images of **a, b** Co/MCCNFs-D and **c, d** Co/IMCCNFs

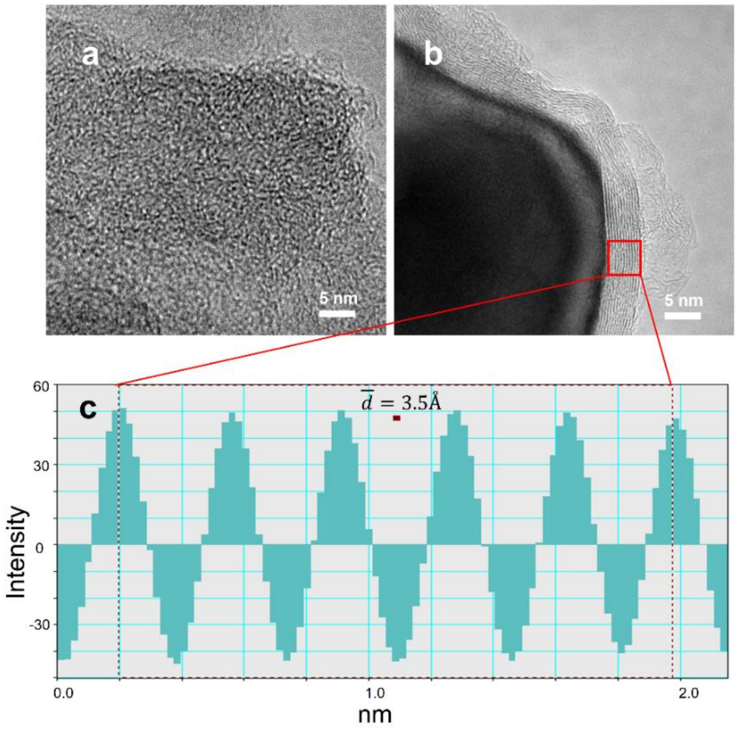

Fig. S3 High-resolution TEM images of **a** MCCNFs and **b** Co/IMCCNFs; **c** Lattice-distance profile of the red frame in the image **b**

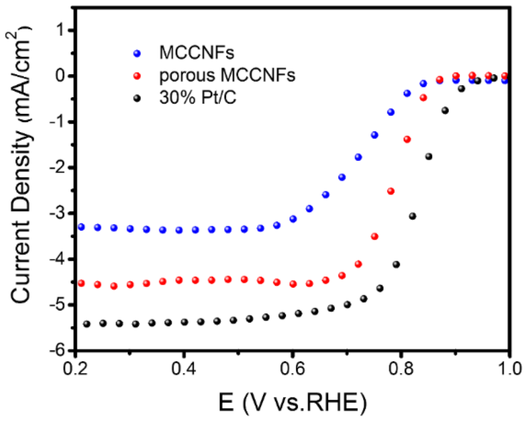

Fig. S4 Rotating-disk voltammograms of 30% Pt/C, MCCNFs and porous MCCNFs in 0.1 M KOH

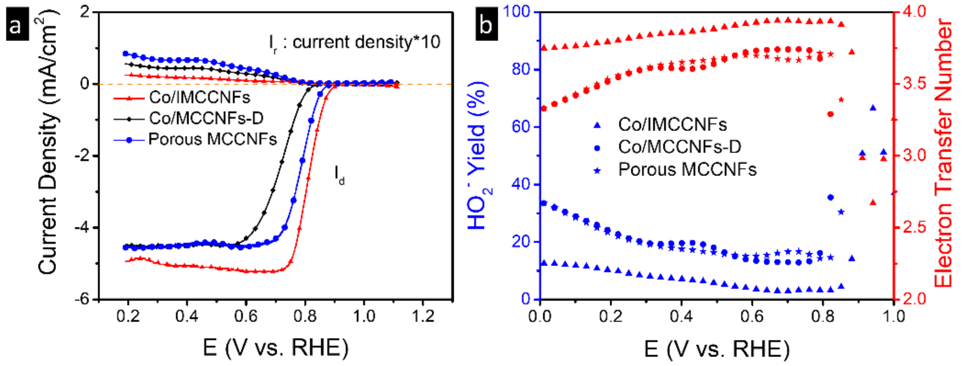

Fig. S5 a Rotating ring-disk electrode voltammograms recorded with porous MCCNFs, Co/IMCCNFs and Co/MCCNFs-D. **b** Percentage of peroxide (lower line) and the electron transfer number (*n*) (upper line) of porous MCCNFs, Co/IMCCNFs, and Co/MCCNFs-D at various potentials, based on the corresponding RRDE data in **a**

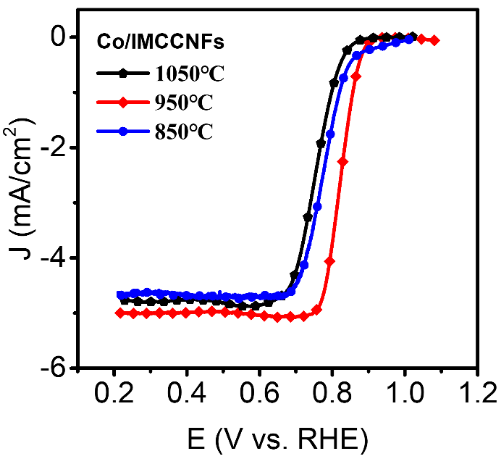

Fig. S6 Rotating-disk voltammograms of Co/IMCCNFs which were pyrolyzed at different temperatures
